# Carotid Intima-Media Thickness in Chronic Kidney Disease: Evidence of Early Vascular Injury in a Tertiary Care Cohort in Northeast India

**DOI:** 10.7759/cureus.100057

**Published:** 2025-12-25

**Authors:** Aswath R Deepa, Anish Ancil, Ameena A Jaleel, Lydia M Abraham, Tasyoh Thampi, Arooj Zainab, Debashrita Baidya

**Affiliations:** 1 Department of Internal Medicine, Agartala Government Medical College & Govind Ballabh Pant Hospital, Agartala, IND; 2 Department of Pharmacology, Agartala Government Medical College & Govind Ballabh Pant Hospital, Agartala, IND; 3 Department of Internal Medicine, Travancore Medical College Hospital, Kollam, IND; 4 Department of Internal Medicine, VPS Lakeshore Hospital, Kochi, IND; 5 Department of Internal Medicine, Sunrise Hospital Kochi, Kochi, IND; 6 Department of Biology, Wofford College, Spartanburg, USA

**Keywords:** atherosclerosis, cardiovascular risk, carotid intima-media thickness (cimt), chronic kidney disease (ckd), subclinical vascular disease, surrogate marker, ultrasonography

## Abstract

Background

Chronic kidney disease (CKD) is associated with a markedly increased cardiovascular risk that is often underestimated by traditional risk prediction models. Carotid intima-media thickness (CIMT) is a noninvasive marker of subclinical vascular injury, but data from Northeast India remain limited.

Methods

We conducted a cross-sectional study of 160 adults with CKD stages I to V attending a tertiary care center in Northeast India. Patients with a prior history of myocardial infarction, stroke, or vascular revascularization were excluded. CIMT was measured bilaterally using high-resolution B-mode ultrasonography, with three measurements obtained on each side and averaged to derive a participant-level mean. Abnormal CIMT was defined a priori as greater than 0.9 mm. Associations between CIMT and clinical variables were evaluated using multivariable linear regression adjusted for age, sex, CKD stage, hemodialysis status, hypertension, diabetes mellitus, and smoking.

Results

The mean age of participants was 40.3 ± 11.7 years, and 71.3% were male. Stage V CKD was the most common stage, accounting for 40.6% of patients. The overall mean CIMT was 1.04 ± 0.26 mm, and abnormal values were present in 122 of 160 participants (76.3%). Mean CIMT exceeded 0.9 mm by stage IIIa CKD. Patients receiving hemodialysis had significantly higher CIMT compared with those not on hemodialysis. In adjusted analyses, more advanced CKD stage, hemodialysis status, hypertension, and older age were independently associated with higher CIMT, while sex, diabetes mellitus, and smoking were not.

Conclusions

In this relatively young CKD cohort, a high burden of subclinical vascular injury was observed, with clinically significant carotid wall thickening detectable by stage IIIa. These findings support the use of CIMT as a practical adjunct for cardiovascular risk assessment in patients with CKD in resource-constrained settings.

## Introduction

Chronic kidney disease (CKD) is a common and progressive condition that affects more than 10% of adults worldwide [1]. In India, the estimated prevalence is higher, at approximately 17%, largely driven by hypertension and diabetes mellitus [2,3]. Patients in India frequently present at a comparatively young age and at more advanced stages of CKD, which narrows the window for preventive intervention and limits opportunities to reduce long-term cardiovascular risk [3,4].

Cardiovascular disease is the leading cause of death among individuals with CKD, with event rates several-fold higher than those observed in the general population [5]. This excess risk reflects the combined influence of traditional cardiovascular risk factors, including hypertension, dyslipidemia, diabetes, and tobacco use, as well as CKD-related biological mechanisms such as chronic inflammation, oxidative stress, endothelial dysfunction, medial arterial calcification, and retention of uremic solutes [6]. Vascular disease in CKD is therefore characterized by both classic intimal atherosclerosis driven by lipid deposition and nonclassic medial calcification related to disordered mineral metabolism, resulting in early and diffuse structural arterial injury. Conventional cardiovascular risk prediction tools, including those derived from the Framingham cohort, were developed in populations without significant renal impairment and do not account for these CKD-specific pathophysiological processes. Consequently, such tools tend to underestimate cardiovascular risk in patients with CKD [7].

Carotid intima-media thickness (CIMT), measured noninvasively using high-resolution B-mode ultrasonography, is a well-established marker of subclinical arterial injury and vascular remodeling [8]. CIMT values greater than 0.9 mm are commonly interpreted as indicating increased vascular risk [8]. In patients with CKD, CIMT has been shown to progress more rapidly than in the general population [9], and higher values are associated with increased rates of myocardial infarction and stroke, particularly among those receiving maintenance hemodialysis [10]. Evidence from large CKD cohorts further suggests that CIMT provides prognostic information for both prevalent and incident cardiovascular disease [11].

Despite this growing body of evidence, contemporary data from Northeast India remain scarce. The regional clinical profile, characterized by younger patients, late-stage presentation, and frequent reliance on hemodialysis, suggests a substantial burden of unrecognized subclinical vascular injury [3,4]. At the same time, carotid ultrasonography is widely available and relatively inexpensive in public-sector hospitals, making CIMT a practical and accessible bedside tool for cardiovascular risk assessment in resource-constrained settings.

We therefore conducted a cross-sectional study of adults with CKD stages I through V at a tertiary care center in Northeast India. Patients with known cardiovascular disease were excluded to enable focused evaluation of subclinical arterial injury. The objectives of this study were to determine the prevalence of abnormal CIMT, defined a priori as greater than 0.9 mm, to identify the stage of CKD at which mean CIMT first exceeds this high-risk threshold, and to examine clinical correlates of CIMT, including CKD stage, hemodialysis status, hypertension, diabetes mellitus, smoking, and age, in a relatively young population with a high burden of renal disease.

## Materials and methods

Study design and setting

We conducted a cross-sectional study among adult patients with CKD receiving care at a tertiary care hospital in Northeast India. Consecutive eligible participants with CKD stages I through V were enrolled during routine nephrology outpatient visits. The study protocol was approved by the Institutional Ethics Committee, and written informed consent was obtained from all participants prior to enrollment.

Eligibility criteria

Adults with established CKD were eligible for inclusion. CKD stage was determined using the estimated glomerular filtration rate calculated with the CKD-EPI equation [12] and classified according to Kidney Disease: Improving Global Outcomes (KDIGO) 2012 criteria [13]. To focus specifically on subclinical arterial injury, patients with known clinical cardiovascular disease were excluded. Exclusion criteria included a documented history of myocardial infarction, stroke, or coronary or peripheral vascular revascularization.

Clinical variables

For each participant, demographic and clinical characteristics were recorded, including age, sex, CKD stage, and hemodialysis status at the time of assessment. The presence or absence of hypertension, diabetes mellitus, and smoking was documented based on clinical evaluation and review of medical records:

Hypertension was defined as a documented diagnosis of hypertension, current use of antihypertensive medication, or a systolic blood pressure ≥140 mmHg or diastolic blood pressure ≥90 mmHg on repeated clinical measurements. Diabetes mellitus was defined as a documented diagnosis, current use of glucose-lowering medication, or laboratory criteria consistent with diabetes, including fasting plasma glucose ≥126 mg/dL, random plasma glucose ≥200 mg/dL, or glycated hemoglobin ≥6.5%. Smoking status was categorized as current or former smoker versus never smoker.

All data were collected using a standardized data abstraction form to ensure uniformity and reduce information bias.

CIMT assessment

CIMT was measured using high-resolution B-mode ultrasonography. Both common carotid arteries were examined in longitudinal view, with assessment of the far wall approximately 1 cm proximal to the carotid bifurcation. Three measurements were obtained from each side and averaged. Participant-level CIMT was defined as the mean of six measurements, three from the right carotid artery and three from the left.

All examinations were performed using the same ultrasound system with uniform probe frequency, depth, gain, and insonation settings to minimize measurement variability. A CIMT value >0.9 mm was defined a priori as abnormal and indicative of increased vascular risk, consistent with established vascular ultrasound literature [8].

Outcomes

The primary outcome was the prevalence of abnormal CIMT, defined as a value greater than 0.9 mm. Secondary outcomes included the mean CIMT across CKD stages to identify the earliest stage at which the average CIMT exceeded this threshold and the associations between CIMT and prespecified clinical variables, including age, sex, CKD stage, hemodialysis status, hypertension, diabetes mellitus, and smoking status.

Sample size

Sample size was determined using a precision-based approach appropriate for estimating a single proportion in a descriptive cross-sectional study. Assuming a prevalence of abnormal CIMT of 50%, selected to maximize variance and yield a conservative estimate, and targeting an absolute precision of 0.08 with a two-sided 95% confidence level, the minimum required sample size was approximately 150 participants. A total of 160 participants were enrolled to account for potential measurement variability and to ensure representation across all CKD stages.

Statistical analysis

Continuous variables are presented as mean ± SD, and categorical variables as counts and percentages. Mean CIMT values were compared across categories defined by age group, sex, hemodialysis status, hypertension, diabetes mellitus, smoking status, and CKD stage. For the CKD stage, a test for linear trend across ordered categories was performed. To examine independent associations with CIMT, a multivariable linear regression model was fitted with CIMT as the dependent variable. Prespecified covariates entered simultaneously into the model included age, sex, CKD stage, hemodialysis status, hypertension, diabetes mellitus, and smoking status. Standardized beta coefficients with corresponding 95% confidence intervals and p-values were reported, and a two-sided p-value <0.05 was considered statistically significant.

## Results

Study population

A total of 160 adults with CKD were included in the analysis. Baseline characteristics are summarized in Table 1. The mean age of the study population was 40.3 ± 11.7 years, and most participants were male (114 of 160, 71.3%). The cohort was clinically advanced, with CKD stage V being the most frequent stage at presentation (65 of 160 patients, 40.6%), while only 12 patients (7.5%) were classified as stage I.

**Table 1:** Baseline characteristics of the study population (n = 160) CKD, chronic kidney disease; KDIGO, Kidney Disease: Improving Global Outcomes

Characteristic	Value
Age, years	
Mean ± SD	40.3 ± 11.7
Sex, n (%)	
Male	114 (71.3)
Female	46 (28.8)
CKD stage (KDIGO), n (%)	
Stage I	12 (7.5)
Stage II	25 (15.6)
Stage IIIa	26 (16.3)
Stage IIIb	21 (13.1)
Stage IV	11 (6.9)
Stage V	65 (40.6)
Comorbid conditions, n (%)	
Hypertension	108 (67.5)
Diabetes mellitus	93 (58.1)
Smoking (current or former)	103 (64.4)
Receiving hemodialysis, n (%)	97 (60.6)

Comorbid conditions were common. Hypertension was present in 108 of 160 participants (67.5%), diabetes mellitus in 93 of 160 (58.1%), and current or former smoking in 103 of 160 (64.4%). At the time of assessment, 97 of 160 participants (60.6%) were receiving maintenance hemodialysis.

CIMT

CIMT measurements are presented in Table 2. The mean right-sided CIMT was 1.04 ± 0.26 mm, and the mean left-sided CIMT was 1.05 ± 0.28 mm. The overall mean CIMT, defined as the average of six measurements per participant, was 1.04 ± 0.26 mm. Using the predefined threshold of >0.9 mm, abnormal CIMT was identified in 122 of 160 participants (76.3%). Thus, approximately three-quarters of the study population demonstrated carotid wall thickening consistent with increased vascular risk.

**Table 2:** CIMT measurements (n = 160) Overall mean CIMT was calculated as the mean of six measurements per participant (three on the right side and three on the left side). CIMT, carotid intima-media thickness

Measurement	Value (mm ± SD)
Right CIMT	1.04 ± 0.26
Left CIMT	1.05 ± 0.28
Overall mean CIMT	1.04 ± 0.26

Age, clinical factors, and CIMT

**Figure 1:**
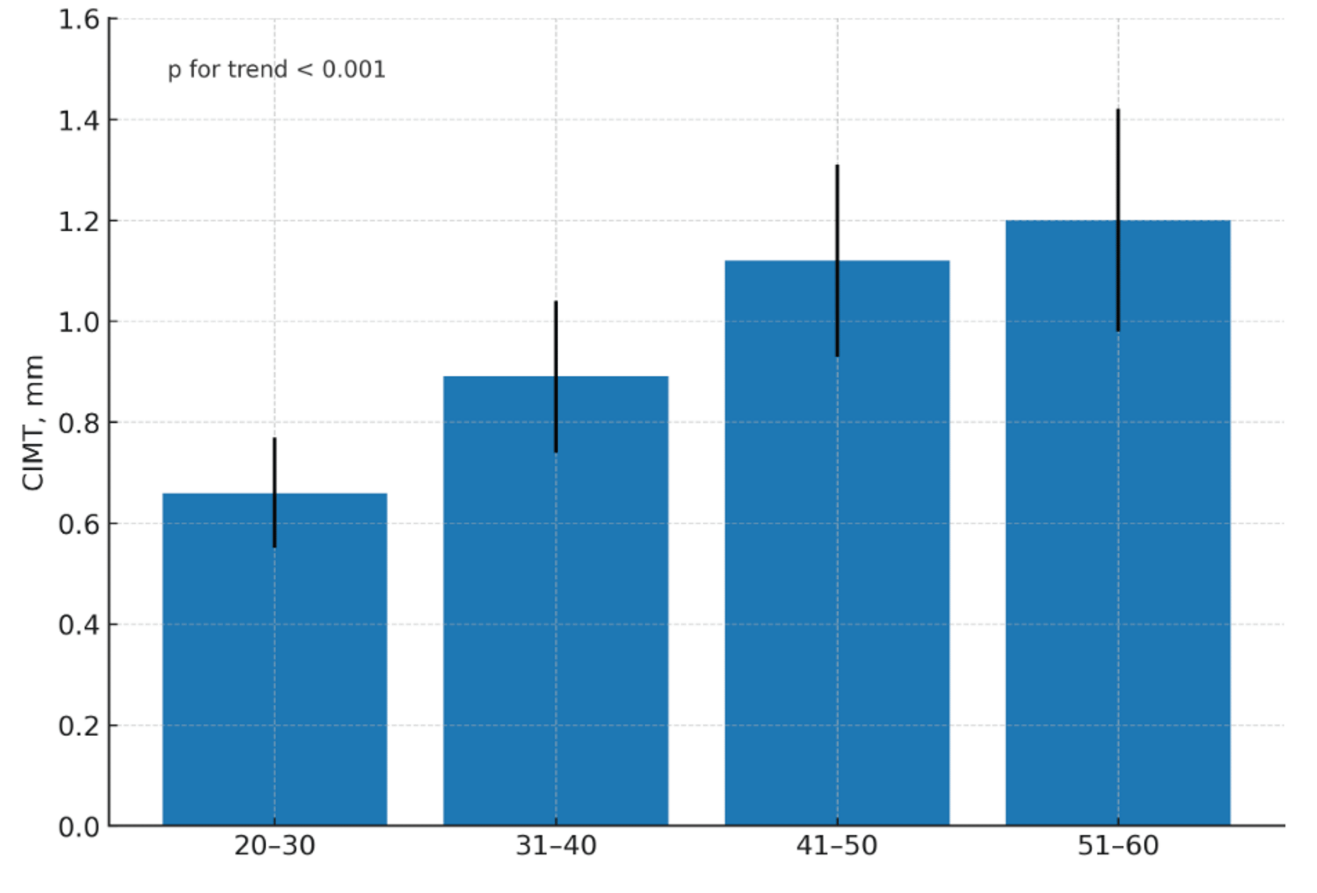
Mean CIMT by age group Bars represent mean CIMT with SD in patients aged 20-30, 31-40, 41-50, and 51-60 years. The p-value for linear trend across age groups is <0.001. CIMT, carotid intima-media thickness

Variation in CIMT across demographic and clinical strata is shown in Table 3. CIMT increased progressively with advancing age, from a mean of 0.66 ± 0.11 mm in participants aged 20-30 years to 1.20 ± 0.22 mm in those aged 51-60 years (p for trend < 0.001). This graded association is illustrated in Figure 1. Mean CIMT values did not differ significantly between men and women (1.07 ± 0.27 mm vs. 1.05 ± 0.24 mm, p = 0.21).

**Table 3:** CIMT across demographic and clinical strata Significant p-values are indicated with “^*^”. Statistical significance was defined as p < 0.05 (highly significant at p < 0.001). A hyphen (-) denotes that no p-value applies for that row. CIMT, carotid intima-media thickness; CKD, chronic kidney disease; KDIGO, Kidney Disease: Improving Global Outcomes

Stratum	Category	Mean CIMT (mm ± SD)	p-Value
Age group (years)	20-30	0.66 ± 0.11	-
	31-40	0.89 ± 0.15	-
	41-50	1.12 ± 0.19	-
	51-60	1.20 ± 0.22	-
	P for trend	-	<0.001^*^
Sex	Male	1.07 ± 0.27	-
	Female	1.05 ± 0.24	0.21
Hemodialysis	Yes	1.23 ± 0.25	<0.001^*^
	No	0.76 ± 0.18	-
Hypertension	Present	1.19 ± 0.25	<0.001^*^
	Absent	0.74 ± 0.16	-
Diabetes mellitus	Present	1.21 ± 0.26	0.001^*^
	Absent	0.82 ± 0.18	-
Smoking status	Current or former	1.18 ± 0.26	0.001^*^
	Never	0.81 ± 0.19	-
CKD (KDIGO stage)	Stage I	0.62 ± 0.10	-
	Stage II	0.85 ± 0.14	-
	Stage IIIa	1.07 ± 0.17	-
	Stage IIIb	1.12 ± 0.18	-
	Stage IV	1.15 ± 0.20	-
	Stage V	1.25 ± 0.24	-
	P for trend	-	<0.001^*^

Clinical factors associated with vascular remodeling were strongly related to CIMT in unadjusted analyses. Participants receiving hemodialysis had substantially higher CIMT values compared with those not on hemodialysis (1.23 ± 0.25 mm vs. 0.76 ± 0.18 mm, p < 0.001). CIMT was also higher among participants with hypertension (1.19 ± 0.25 mm vs. 0.74 ± 0.16 mm, p < 0.001), those with diabetes mellitus (1.21 ± 0.26 mm vs. 0.82 ± 0.18 mm, p = 0.001), and current or former smokers compared with never smokers (1.18 ± 0.26 mm vs. 0.81 ± 0.19 mm, p = 0.001).

CIMT and CKD stage

Mean CIMT increased steadily with worsening CKD stage. Mean values were 0.62 ± 0.10 mm in stage I, 0.85 ± 0.14 mm in stage II, 1.07 ± 0.17 mm in stage IIIa, 1.12 ± 0.18 mm in stage IIIb, 1.15 ± 0.20 mm in stage IV, and 1.25 ± 0.24 mm in stage V (p for trend < 0.001).

A notable threshold was observed at CKD stage IIIa, at which the mean CIMT exceeded 0.9 mm. This indicates that carotid wall thickening consistent with increased vascular risk was detectable before progression to stage IV and prior to the dialysis-dependent stage V.

Multivariable analysis

In the prespecified multivariable linear regression model, four variables remained independently associated with higher CIMT after adjustment for all covariates. More advanced CKD stage (standardized beta 0.42, p < 0.001), current hemodialysis status (standardized beta 0.28, p < 0.001), hypertension (standardized beta 0.25, p < 0.001), and older age (standardized beta 0.19, p = 0.008) were significantly associated with increased CIMT. After multivariable adjustment, sex, diabetes mellitus, and smoking status were not independently associated with CIMT.

Taken together, these results highlight three principal observations. First, carotid wall thickening was highly prevalent in this cohort, with more than three-quarters of participants demonstrating abnormal CIMT values. Second, structural vascular changes were detectable at relatively early stages of CKD, with mean CIMT exceeding the high-risk threshold by stage IIIa in a population with a mean age of 40 years. Third, increased CIMT reflected the combined influence of kidney disease severity and cumulative hemodynamic burden, with CKD stage, hemodialysis status, hypertension, and age showing independent associations.

## Discussion

This cross-sectional study of 160 adults with CKD from a tertiary care center in Northeast India demonstrates a substantial burden of structural carotid arterial injury. The mean CIMT was elevated at 1.04 ± 0.26 mm, with 76.3% of participants (n = 122) exceeding the 0.9 mm threshold commonly associated with increased vascular risk [8,11]. Importantly, this degree of subclinical atherosclerosis was observed in a relatively young cohort with a mean age of approximately 40 years, most of whom had no documented history of myocardial infarction, stroke, or vascular revascularization. This finding highlights a marked disconnect between traditional cardiovascular risk perception and the accelerated vascular injury that characterizes CKD [5]. The predominance of advanced disease at presentation, with 40.6% of participants in stage V CKD, aligns with regional reports of delayed diagnosis and underscores the heightened cardiovascular vulnerability in this population [3,4].

A key observation was the clear and graded increase in CIMT across advancing CKD stages, rising from 0.62 mm in stage I to 1.25 mm in stage V [11]. This biological gradient supports the role of CKD-specific pathophysiological processes in vascular injury, beyond the influence of age or conventional risk factors alone. The uremic milieu in CKD is characterized by endothelial dysfunction, chronic low-grade inflammation, oxidative stress, and retention of uremic solutes, all of which promote intimal thickening and vascular remodeling [6,14]. Disturbances in mineral and bone metabolism, particularly phosphate excess, further contribute by accelerating medial arterial calcification and increasing arterial stiffness [15]. Additional contributors, such as chronic volume overload, anemia-related high-output states, and accumulation of proinflammatory middle-molecular-weight toxins, may further compound vascular injury [6,14,15]. Notably, mean CIMT exceeded the 0.9 mm threshold by stage IIIa, indicating that clinically relevant vascular damage is detectable well before progression to end-stage renal disease.

Participants receiving maintenance hemodialysis constituted a distinct high-risk subgroup. Their mean CIMT was 1.23 mm compared with 0.76 mm among participants not receiving dialysis. While reduced kidney function itself is associated with vascular injury, hemodialysis introduces additional physiological stressors, including recurrent intradialytic volume shifts, abrupt changes in intravascular pressure, exposure to bioincompatible membranes, and sustained systemic inflammation [16,17]. These dialysis-related factors may amplify preexisting vascular injury and help explain the more advanced arterial thickening observed in this subgroup.

Traditional cardiovascular risk factors also showed important associations. Hypertension, diabetes mellitus, and smoking were each associated with higher CIMT in unadjusted analyses, with hypertension demonstrating a particularly strong association consistent with chronic pressure-mediated arterial wall remodeling [6]. In the multivariable model, after adjustment for age, sex, CKD stage, hemodialysis status, hypertension, diabetes mellitus, and smoking, four variables remained independently associated with higher CIMT: more advanced CKD stage, current hemodialysis, hypertension, and older age. Diabetes mellitus, smoking status, and sex were no longer independently associated. These findings suggest two intersecting pathways contributing to arterial remodeling in CKD. One reflects cumulative hemodynamic stress, represented by hypertension and advancing age. The other reflects kidney-specific pathophysiology and treatment-related factors, including declining filtration capacity and exposure to hemodialysis [6,14-17].

The clinical implications of these findings are significant. Cardiovascular prevention strategies in CKD are often intensified only after dialysis initiation or following a cardiovascular event, despite evidence that conventional population-based risk scores underestimate risk in CKD [7]. CIMT measurement using standardized B-mode ultrasonography is a noninvasive, accessible, and reproducible method for assessing subclinical vascular injury [8]. Identification of a CIMT value greater than 0.9 mm in a patient with stage III CKD and no prior cardiovascular events may provide objective evidence of established vascular disease and support earlier intensification of preventive strategies. Such strategies include optimized lipid-lowering therapy [18], effective blood pressure control using regimens that support both renal and vascular health [19], smoking cessation, and appropriate glycemic management when applicable.

The vascular profile observed in this cohort reinforces the concept of CKD as a state of accelerated systemic vascular aging rather than a condition limited to impaired filtration [20,21]. Participants in their third and fourth decades of life demonstrated arterial characteristics more typically seen in much older individuals with high cardiovascular risk. In resource-constrained settings, such as many regions of India, where access to nephrology care often occurs late due to systemic and socioeconomic barriers [3,4,22], CIMT may serve as a pragmatic bedside tool to identify high-risk individuals who could benefit from earlier and more intensive cardiovascular risk reduction.

This study has several strengths, including the use of a standardized CIMT measurement protocol with bilateral assessment and averaging of six measurements per participant, inclusion of participants across all KDIGO stages, and generation of region-specific data from Northeast India, an area underrepresented in CKD and cardiovascular research [3,4,22]. However, certain limitations should be acknowledged. The cross-sectional design precludes causal inference and assessment of future cardiovascular outcomes. The single-center, hospital-based sampling frame enriched for advanced CKD may limit generalizability to community-detected or earlier-stage disease. In addition, data on lipid profiles, inflammatory biomarkers, mineral metabolism parameters, and dialysis vintage were not uniformly available, limiting mechanistic interpretation [14-20]. Finally, exclusion of participants with known cardiovascular disease was intentional to focus on subclinical injury but likely resulted in underestimation of the overall burden of carotid pathology in the broader CKD population.

## Conclusions

In this cohort of adults with CKD from Northeast India, a high burden of subclinical carotid atherosclerosis was observed, with increasing CIMT closely associated with worsening renal dysfunction. Clinically relevant arterial thickening was detectable by CKD stage IIIa, indicating that vascular injury occurs early in the disease course. These findings support the use of CIMT measurement as a practical adjunct to cardiovascular risk assessment in patients with CKD, particularly in resource-constrained settings. Early identification of elevated CIMT may facilitate the timely implementation of guideline-directed preventive strategies aimed at reducing the excess cardiovascular risk associated with CKD.

## References

[1] Jager KJ, Kovesdy C, Langham R, Rosenberg M, Jha V, Zoccali C (2019). A single number for advocacy and communication-worldwide more than 850 million individuals have kidney diseases. Kidney Int.

[2] Singh AK, Farag YM, Mittal BV (2013). Epidemiology and risk factors of chronic kidney disease in India - results from the SEEK (Screening and Early Evaluation of Kidney Disease) study. BMC Nephrol.

[3] Rajapurkar MM, John GT, Kirpalani AL (2012). What do we know about chronic kidney disease in India: first report of the Indian CKD registry. BMC Nephrol.

[4] Agarwal SK, Srivastava RK (2009). Chronic kidney disease in India: challenges and solutions. Nephron Clin Pract.

[5] Sarnak MJ, Levey AS, Schoolwerth AC (2003). Kidney disease as a risk factor for development of cardiovascular disease: a statement from the American Heart Association Councils on Kidney in Cardiovascular Disease, High Blood Pressure Research, Clinical Cardiology, and Epidemiology and Prevention. Hypertension.

[6] Moody WE, Edwards NC, Chue CD, Ferro CJ, Townend JN (2013). Arterial disease in chronic kidney disease. Heart.

[7] Weiner DE, Tighiouart H, Elsayed EF, Griffith JL, Salem DN, Levey AS, Sarnak MJ (2007). The Framingham predictive instrument in chronic kidney disease. J Am Coll Cardiol.

[8] Stein JH, Korcarz CE, Hurst RT (2008). Use of carotid ultrasound to identify subclinical vascular disease and evaluate cardiovascular disease risk: a consensus statement from the American Society of Echocardiography Carotid Intima-Media Thickness Task Force Endorsed by the Society for Vascular Medicine. J Am Soc Echocardiogr.

[9] Shimoyama Y, Tsuruta Y, Niwa T (2012). Coronary artery calcification score is associated with mortality in Japanese hemodialysis patients. J Ren Nutr.

[10] Benedetto FA, Mallamaci F, Tripepi G, Zoccali C (2001). Prognostic value of ultrasonographic measurement of carotid intima media thickness in dialysis patients. J Am Soc Nephrol.

[11] Adeseun GA, Xie D, Wang X (2012). Carotid plaque, carotid intima-media thickness, and coronary calcification equally discriminate prevalent cardiovascular disease in kidney disease. Am J Nephrol.

[12] Levey AS, Stevens LA, Schmid CH (2009). A new equation to estimate glomerular filtration rate. Ann Intern Med.

[13] Levin A, Stevens PE, Bilous RW (2013). Kidney Disease: Improving Global Outcomes (KDIGO) CKD work group. KDIGO 2012 clinical practice guideline for the evaluation and management of chronic kidney disease. Kidney Int Suppl.

[14] Stenvinkel P, Carrero JJ, Axelsson J, Lindholm B, Heimbürger O, Massy Z (2008). Emerging biomarkers for evaluating cardiovascular risk in the chronic kidney disease patient: how do new pieces fit into the uremic puzzle?. Clin J Am Soc Nephrol.

[15] London GM, Guérin AP, Marchais SJ, Métivier F, Pannier B, Adda H (2003). Arterial media calcification in end-stage renal disease: impact on all-cause and cardiovascular mortality. Nephrol Dial Transplant.

[16] McIntyre CW (2010). Haemodialysis-induced myocardial stunning in chronic kidney disease - a new aspect of cardiovascular disease. Blood Purif.

[17] McIntyre CW, Harrison LE, Eldehni MT (2011). Circulating endotoxemia: a novel factor in systemic inflammation and cardiovascular disease in chronic kidney disease. Clin J Am Soc Nephrol.

[18] Wanner C, Krane V, März W, Olschewski M, Mann JF, Ruf G, Ritz E (2005). Atorvastatin in patients with type 2 diabetes mellitus undergoing hemodialysis. N Engl J Med.

[19] Cheung AK, Rahman M, Reboussin DM (2017). Effects of intensive BP control in CKD. J Am Soc Nephrol.

[20] Zoccali C, Vanholder R, Massy ZA (2017). The systemic nature of CKD. Nat Rev Nephrol.

[21] London GM (2018). Arterial stiffness in chronic kidney disease and end-stage renal disease. Blood Purif.

[22] Varughese S, Abraham G (2018). Chronic kidney disease in India: a clarion call for change. Clin J Am Soc Nephrol.

